# A diurnal flux balance model of *Synechocystis* sp. PCC 6803 metabolism

**DOI:** 10.1371/journal.pcbi.1006692

**Published:** 2019-01-24

**Authors:** Debolina Sarkar, Thomas J. Mueller, Deng Liu, Himadri B. Pakrasi, Costas D. Maranas

**Affiliations:** 1 Department of Chemical Engineering, Pennsylvania State University, University Park, Pennsylvania, United States of America; 2 Department of Biology, Washington University, St. Louis, Missouri, United States of America; Humboldt University Berlin, GERMANY

## Abstract

Phototrophic organisms such as cyanobacteria utilize the sun’s energy to convert atmospheric carbon dioxide into organic carbon, resulting in diurnal variations in the cell’s metabolism. Flux balance analysis is a widely accepted constraint-based optimization tool for analyzing growth and metabolism, but it is generally used in a time-invariant manner with no provisions for sequestering different biomass components at different time periods. Here we present CycleSyn, a periodic model of *Synechocystis* sp. PCC 6803 metabolism that spans a 12-hr light/12-hr dark cycle by segmenting it into 12 Time Point Models (TPMs) with a uniform duration of two hours. The developed framework allows for the flow of metabolites across TPMs while inventorying metabolite levels and only allowing for the utilization of currently or previously produced compounds. The 12 TPMs allow for the incorporation of time-dependent constraints that capture the cyclic nature of cellular processes. Imposing bounds on reactions informed by temporally-segmented transcriptomic data enables simulation of phototrophic growth as a single linear programming (LP) problem. The solution provides the time varying reaction fluxes over a 24-hour cycle and the accumulation/consumption of metabolites. The diurnal rhythm of metabolic gene expression driven by the circadian clock and its metabolic consequences is explored. Predicted flux and metabolite pools are in line with published studies regarding the temporal organization of phototrophic growth in *Synechocystis* PCC 6803 paving the way for constructing time-resolved genome-scale models (GSMs) for organisms with a circadian clock. In addition, the metabolic reorganization that would be required to enable *Synechocystis* PCC 6803 to temporally separate photosynthesis from oxygen-sensitive nitrogen fixation is also explored using the developed model formalism.

## Author summary

Phototrophic organisms such as cyanobacteria harvest the sun’s energy to convert atmospheric CO_2_ into organic carbon, due to which their metabolism is heavily influenced by light availability. The strongly diurnal nature of their metabolism is reflected in the presence of two distinct metabolic phases–a light-dependent anabolic phase tailored around the synthesis of storage compounds and metabolic precursors and a light-absent catabolic period that metabolizes the previously manufactured compounds to release energy in the absence of an external energy source. Due to these considerations, the analysis of phototrophic growth using constraint-based optimization methods is insufficient and needs to be extended beyond time-invariant descriptions. Here, we introduce CycleSyn which models the periodic nature of metabolism in *Synechocystis* sp. PCC 6803. Our approach enables us to account for temporal metabolic shifts tailored around light availability while still allowing for the use of the pseudo steady-state assumption used in conventional flux balance analysis. This is achieved by exploiting the large difference in time-scales between metabolic reactions and cell growth. We first validate the biological fidelity of CycleSyn predictions by comparing them to experimental observations for a diurnally cultured *Synechocystis* sp. PCC 6803 and to identify the major temporal variations in its metabolic processes. Next, we demonstrate the ability of CycleSyn to describe a temporally-varying metabolism by introducing diazotrophy in *Synechocystis* and evaluating the genes that need to be upregulated/downregulated to enable nitrogen fixation in a photosynthetic organism. Our study lays the foundation for subsequent analysis of systems with temporal variations in metabolism using a constraint-based optimization approach.

## Introduction

Flux balance analysis (FBA) has become a popular tool to analyze the metabolic function of organisms [1]. FBA assumes the cell is operating at a pseudo steady-state, wherein for each internal metabolite the sum of production fluxes must equal the sum of consumption fluxes. The steady-state assumption hinges upon the requirement that the time constants characterizing metabolic reactions are very rapid compared to the time constant of cell growth [2]. This time flux invariance places tight constraints on the feasible solution space and underpins the explanatory and predictive success of FBA [3–5]. However, for many organisms temporal and periodic variations in metabolism are part of their lifestyle [6]. This is the case for phototrophic organisms whose metabolism is tailored around light availability over a 24-hour cycle. Two distinct phases can be identified here: a light phase that centers around synthesis of metabolic precursors and storage compounds, and a dark phase that consumes those storage compounds to ensure survival in the absence of an energy source [7]. The transition between these two phases is driven by the circadian clock that choreographs the temporal expression of thousands of genes [6]. Highly varying gene expression levels over the 24hr cycle implies that the corresponding metabolic fluxes would also vary significantly and the biomass precursor production be dynamically shaped as the cumulative contribution by metabolism over 24 hours. FBA describes metabolic fluxes as the average over the 24hr period thus missing the opportunity to describe the (i) temporally varying nature of metabolism, (ii) time dependent inventory and remobilization of metabolites, and (iii) the time when different components of biomass are produced. This implies that FBA needs to be augmented so that it can accommodate temporally varying gene transcription information while still permitting the use of the pseudo steady-state assumption, by exploiting the difference in time-scales between metabolic reactions and cell growth.

In their natural habitat, cyanobacteria are subject to a diurnal cycle of light and dark, leading to significant shifts and reorganization within their metabolic network. Although several studies, both experimental and computational [8–10], have helped to illustrate this cyclic cyanobacterial lifestyle, metabolic studies have primarily focused on conditions of constant illumination or heterotrophic growth on externally-supplied carbon sources. Kinetic models of cyanobacterial metabolism can capture the temporal biochemical interactions in the system, but are only available for select subsystems, such as the cyanobacterial circadian clock [11,12], photosystem II [13], and the Calvin-Benson cycle [14,15]. These temporal transitions cannot be described using conventional FBA, and these limitations have been recognized before. Knoop et al. [16] augmented FBA by introducing a time varying biomass composition tailored around light availability. For instance, the ratio for pigments in the biomass reaction was increased two hours before sunrise and storage compound coefficients increased after noon. A set amount of glycogen was supplied to the model for fueling dark respiration instead of transferring the storage compounds generated during light to dark [16]. Optimal temporal allocation of resources has also been employed as a tool to model diurnal lifestyles using an approach called conditional FBA [17,18]. Conditional FBA limits the flux through a reaction by accounting for the abundance of enzymes (or enzyme complexes) and their catalytic turnover numbers [17,18]. Nonlinear constraints are used to maintain periodicity which makes the size of the model grow as the square of the number of time steps being simulated [17,18]. The LP problems solved at each iteration may also become ill-conditioned due to the orders of magnitude differences in fluxes. Finally, required inputs such as enzyme turnovers are often hard to determine accurately.

CycleSyn alleviates these challenges by recasting diurnal growth as a single linear optimization framework, with solve times of the same order as that of a standard FBA. A 12h light/12h dark cycle is discretized into twelve intervals, each of which abides by the pseudo-steady state hypothesis, to provide twelve snapshots of metabolism (see Table 1 for a comparison of CycleSyn with other published models of dynamic metabolism). By connecting these snapshots by metabolite transfer reactions, CycleSyn also provides insights into metabolite accumulation and consumption which are in line with published literature [19,20]. The only model input apart from model stoichiometry and biomass composition is transcriptomic data collected at 2-hour intervals, which is used to throttle back the upper bounds of corresponding reactions. Apart from modeling phototrophic metabolism, CycleSyn can be applied to functions typically served by conventional FBA such as testing *in silico* knockout mutants or identifying essential reactions/pathways under a varying light regime. In the absence of omics data from mutant strains, algorithms such as RELATCH [21] can be used in conjunction with CycleSyn to predict the effect of gene knockouts. The primary assumption employed there is that perturbed strains minimize relative metabolic changes and increase the capacity of previously active and inactive pathways in order to adapt to perturbations. By employing flux and gene expression data from wild-type strains, Kim *et al*. were able to successfully predict flux distributions in genetically and/or environmentally perturbed *E*. *coli*, *S*. *cerevisiae*, and *B*. *subtilis* strains. The current text demonstrates the ability of CycleSyn to guide the redesign of temporally-varying metabolism by identifying the metabolic shifts required to incorporate diazotrophy in a phototroph such as *Synechocystis* sp. PCC 6803 (hereafter referred to as *Synechocystis*).

**Table 1:** A comparison of CycleSyn with existing models of dynamic metabolism. In comparing the published models, standard metabolic inputs of biomass composition and nutrient uptakes were not counted. For Baroukh et al. [26] EFMs refer to elementary flux modes.

Model Inputs	Model Limitations	Model Outputs	Reference
**Temporal biomass compositions & RNA-seq data under different growth conditions**	Does not account for temporal metabolite accumulation	Flux distributions under autotrophic, heterotrophic, and mixotrophic conditions	Zuñiga et al. [22]
**Time-course metabolomics data**	Computationally intensive MILP-based relaxation algorithm, data intensive	Temporal flux distributions	Bordbar et al. [23]
**Parameterized using flux rate of change constraints**	Computationally expensive as NLP and multiple LPs to be solved	Temporal flux distributions and metabolite concentrations	Mahadevan et al. [24]
**Time-varying biomass objective function**	Does not account for temporal metabolite accumulation	Temporal flux distributions	Knoop et al. [16]
**Enzyme turnover numbers**	Nonlinear model constraints, numerical instabilities	Temporal flux distributions and metabolite accumulation	Rügen et al. [17], Reimers et al. [18]
**Tissue-specific biomass composition**	Does not account for temporal metabolite accumulation	Temporal flux distributions	Oliveira Dal’Molin et al. [25]
**Reaction kinetic parameters**	Network decomposition heuristics, exponential increase in EFMs with network size	Temporal flux distributions and metabolite concentrations	Baroukh et al. [26]
**Time-course transcriptomics data**	Data intensive	Temporal flux distributions and metabolite accumulation	CycleSyn

In this paper, we seek to capture the temporal changes in phototrophic metabolism over a diurnal cycle by modelling a 24-hour day as twelve individual Time-Point Models (TPMs), with each TPM spanning a two-hour period during 12 hours of light and 12 hours of dark. The pseudo-steady state assumption of standard FBA is imposed at every TPM. Metabolite balances are imposed at every TPM though accumulation and/or net consumption of metabolites is allowed. Any metabolite surplus in the cytosol or carboxysome is transferred to the next TPM. Metabolite levels are not allowed to drop below zero implying that all metabolite consumption within a TPM must not exceed the surplus provided by the previous TPM and the amount produced in the current TPM. Reaction flux upper bounds are set in proportion to the temporally varying transcriptomic data. The cascade of TPMs satisfies periodicity constraints by matching the output from the 12^th^ TPM with the input to the 1^st^ one. Comparisons with experimental observations for *Synechocystis* cultured under a 12h/12h light-dark cycle are used to ascertain biological fidelity. We find that CycleSyn correctly predicted the accumulation of metabolites such as glycogen, the primary storage compound in *Synechocystis*, and was able to replicate the temporal variations in metabolic pathways as seen in a diurnally cultured *Synechocystis*. We also found that upon constricting reaction fluxes using temporally segmented transcriptomic data, the primary bottlenecks in wild-type *Synechocystis* biomass production centered around pyruvate and 2-oxoglutarate metabolisms. Upregulating their production and/or diverting pyruvate flux selectively into the TCA cycle would lead to increased growth, as has been experimentally observed in *Synechococcus elongatus* PCC 7942 [27].

Subsequently, we used the 24hr model to address the metabolic flux rewiring needed to enable nitrogen fixation in a temporally segregated manner from photosynthesis in *Synechocystis*. We found that the added energy needed to fuel nitrogen fixation needs to be supplied by an enhanced TCA cycle turnover together with an upregulation of photosynthesis and glycolysis. The genes that need to be upregulated with respect to a non-diazotrophic wild-type *Synechocystis* are associated with pathways of energy metabolism, so as to meet the higher energy requirements posed by nitrogen fixation and amino-acid production.

## Results/Discussion

### Model structure

**Fig 1:**
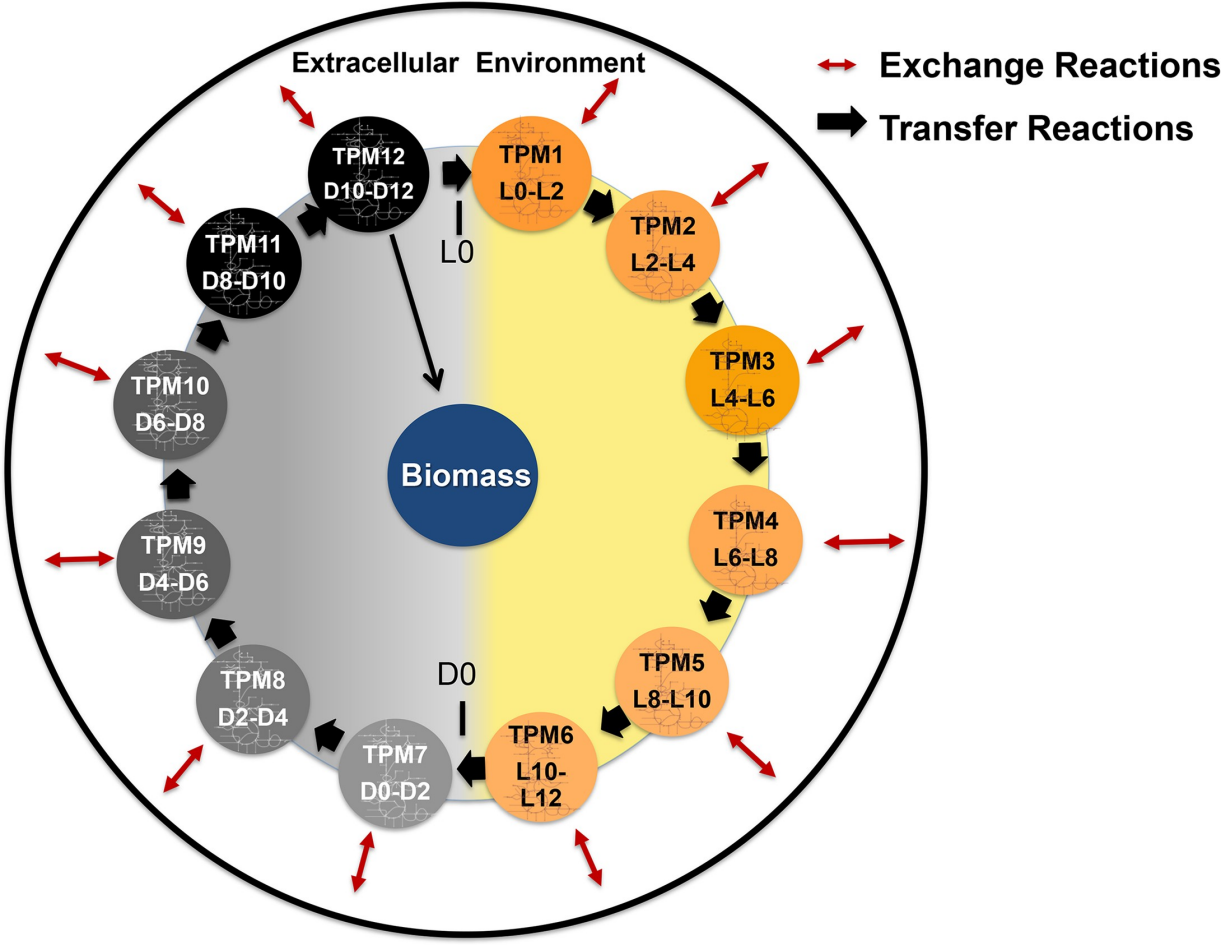
An overview of CycleSyn. There are 12 individual TPMs, with metabolites being transferred through them unidirectionally in the direction indicated. Photons are only supplied to the first six TPMs, and biomass is sequestered in TPM12, i.e., the last dark TPM. Exchange reactions move nutrients and other metabolites in and out of the cell, while transfer reactions shuttle metabolites from one TPM to the next.

The 24-hour model was created starting from the published *Synechocystis* genome-scale model (GSM) *i*Syn731 [28] as a reaction source, which was updated to include the latest *Synechocystis* genome annotation from CyanoBase (http://genome.annotation.jp/cyanobase/Synechocystis). Twelve separate GSMs (each called a Time Point Model or TPM) (Fig 1), each spanning a two-hour period starting from the first light time point (L0-L2) to the last dark time point (D10- D12) were linked. Initially, all TPMs are the same except that TPMs 1 through 6 are allowed to take up light as photons and carbon as carbon dioxide whereas TPMs 7 through 12 are not. The maximum CO_2_ uptake rate was set to 1.1 mmol CO_2_ g^-1^ dry weight hr^-1^ for every light TPM, as cyanobacteria are known to not uptake carbon during dark [29–32] and no fixation occurs in the dark due to the lack of photons. This CO_2_ uptake flux corresponds 0 to 13 mg^-1^ dry weight hr^-1^ of carbon [33,34]. A basal ATP maintenance demand was also set for every TPM at 10 mmol g^-1^ dry weight h^-1^ [28]. The TPMs are connected by the unidirectional forward transfer of metabolites present in the cytosol and carboxysomes, thus only allowing for the consumption of a metabolite in a specific TPM if the metabolite was previously produced or is produced during the current TPM. Any metabolite surplus in the cytosol and carboxysome except photons and protons are transferred to the next TPM (see Materials and Methods). The cyclic topology of the TPMs implies that metabolic flux can go around a closed loop forming a thermodynamically infeasible cycle [35]. To remedy this, the sum of flux through all transfer reactions (between TPMs) is minimized using modified parsimonious flux balance analysis (pFBA) [36] after constraining the biomass production flux to its theoretical maximum. This is implemented in CycleSyn as an additional model constraint. It should be noted that even though the transfer of energy metabolites such as ATP, NAD(P), and NAD(P)H is allowed, CycleSyn results retain their qualitative trends when their transfer fluxes were set to zero. This is because transferring a single storage molecule such as glycogen to satisfy energy demands during the dark phase is preferred by CycleSyn as it is more in line with the model constraint of minimizing the sum of all transfer fluxes.

Photosynthesis in the model is coupled to chlorophyll availability by only allowing flux through photosynthesis reactions in a TPM if chlorophyll is present in that TPM, as coupling chlorophyll production to photosynthetic flux places additional demands on chlorophyll synthesis outside of serving as a biomass precursor. This additional demand is corroborated by matching predicted photosynthetic oxygen evolution flux to experimental values (1) (see Materials and Methods). A single biomass sink placed in the last dark TPM (i.e., TMP12) sequesters all biomass components in the experimentally measured ratios to model growth, although CycleSyn results were similar when the biomass drain was placed in TPM6. TPM6 was chosen as a test case as it is known that very little biomass is produced in the dark [37]. It is important to note that the production of different biomass components is apportioned in a non-uniform manner over the twelve TPMs and only in the last TPM are their fluxes combined to form biomass (Fig 1). The periodicity in metabolism is captured by using transcriptomic data collected over two-hour intervals to constraint reaction fluxes [38]. Specifically, the upper flux bound of a reaction was scaled as a function of its associated gene expression value normalized by the maximum expression over all TPMs, thereby constricting the maximum allowable flux through it. Each reaction’s unscaled flux bounds are determined using only stoichiometric and thermodynamic constraints in order to determine the largest feasible flux range (see Materials and Methods). This approach is often referred to as the valve approach of regulation [39]. The predicted biomass production flux before adding transcriptomic constraints was 0.03 hr^-1^ which corresponds to an average doubling time of 22.8 hours [40]. This closely aligns with the experimentally determined doubling time of wild-type *Synechocystis* under phototrophic conditions, which is approximately 24 hours (~0.0288 hr^-1^) [28]. Following the scaling of reaction fluxes using their corresponding transcriptomic ratios, the maximum biomass production flux was reduced by a tenth of its original unconstrained value. This corresponds to a doubling time of ~25 hours. Doubling times in the range of 20–40 hours have been seen for diurnally cultured *Synechocystis* [41].

CycleSyn uses reaction bounds informed from transcriptomic data to distinguish among the individual light and dark TPMs by relatively throttling reaction upper bounds based on gene expression. Implicit to this is the assumption that RNA levels track protein levels. This correlation between gene expression and protein levels has been shown both experimentally and computationally before [42–45]. Notably Zelezniak et al. [46] found that the correlations between gene expression and metabolite concentrations increases when considered against a background of a metabolic network. We assessed the effect of introducing normally-distributed white noise (within one standard deviation of the mean of the gene expression normalized over time) in the connection between gene expression and proteins. We found that CycleSyn predictions were within 19.28% of their unperturbed flux values, when averaged over all metabolites and all time points.

### Temporal variations in metabolite levels

**Fig 2:**
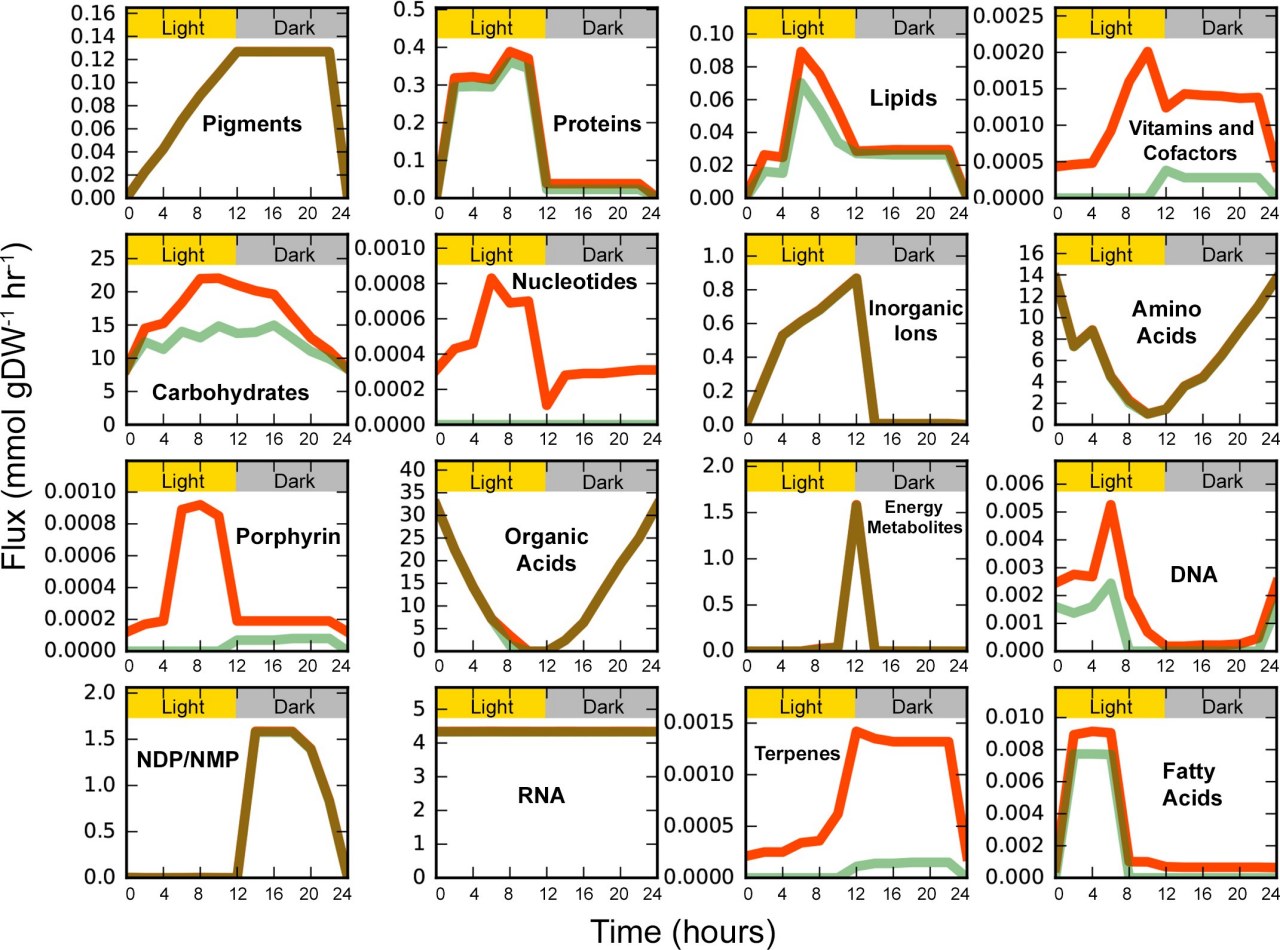
Temporal variations in transfer fluxes of all metabolites over a 24-hour diurnal cycle. Metabolite transfer fluxes were found using flux variability analysis [49], which alternatively minimizes (green) and maximizes (red) flux through the transfer reaction of interest while subject to minimal total flux flow to find the range of feasible reaction fluxes. Individual transfer fluxes were then summed to obtain the total maximum and minimum transfer flux for a category. Category classifications were taken from the KEGG pathway classification database [55].

Different light-sensing proteins help mediate external light cues to coordinate inner metabolic processes. Studies in cyanobacteria such as *Cyanothece* ATCC 51142 (hereafter referred to as *Cyanothece*) have shown that the abundance of many proteins change over the diurnal period [47]. 40.3% of these proteins are associated with central metabolism and energy pathways and 18.5% were associated with photosynthesis and respiration [47]. A majority of *Synechocystis* genes also show cycling, with the peak expression of cycling genes being during the transition from day to night, regulating energy supply and carbon metabolism [48]. Here, we examine if the diurnal nature of a cellular process was maintained from the gene to the metabolic level by incorporating gene expression data using the E-Flux method [39] (see ‘Materials and Methods’ section). Using a modified parsimonious flux balance analysis (pFBA) [36] to minimize the total sum of all fluxes through the metabolite transfers between each TPM, the transfer flux between TPMs was predicted. The transfer flux for a metabolite from one TPM to the next can be interpreted as the accumulation/consumption of that metabolite in that particular TPM. This allows us to compare the model-predicted metabolite accumulation to the experimentally measured metabolite levels in a diurnally cultured *Synechocystis*. As the LP has multiple alternative optima, flux variability analysis [49] was used to determine the maximum and minimum possible transfer flux between TPMs and used to construct Fig 2. This involves minimizing and maximizing the flux through the metabolite transfer reactions while ensuring that the model continues to produce biomass at the maximum value possible and the sum of all transfer fluxes does not exceed the value obtained using the aforementioned modified pFBA. The maximum and minimum transfer flux was taken for every transferring metabolite and summed over to assess the distribution across categories (see supplementary S6 Table for a list of metabolites and their classifications). For metabolite classes such as amino acids, pigments, organic acids, and energy metabolites, there is very little variability between the maximum and minimum transfer flux across TPMs. This is due to the limitations placed on the maximum allowable flux through a reaction using transcriptomics data and the modified pFBA model constraint that further minimizes the sum of all transfer reactions, thus reducing variability. Metabolites are only allowed to accumulate so as to satisfy the demands placed on the system, such as biomass production and ATP maintenance.

The amino acid flux profile, governed mainly by glucogenic amino acids such as proline and alanine, is found to be at its maximum during early light with a sharp decline at the transition between light and dark, i.e. between TPM6 and TPM7. This points towards the role of proline as a carbon and nitrogen reservoir, as is known to occur in Cyanobacteria [50,51]. CycleSyn predicts that proline flux feeds into glutamate biosynthesis during the light TPMs by the action of glutamate dehydrogenase. This is corroborated in literature, where glutamate concentrations are known to increase during light in a diurnally cultured *Synechocystis* [41] and the NADP-specific glutamate dehydrogenase functions only during light in the phototroph *C*. *sorokiniana* [52]. The decrease in amino acid transfer flux during the transition from light to dark can be explained by the concurrent increase in accumulation of energy metabolites such as ATP, GTP and CTP. Glucogenic amino acids are degraded towards TCA cycle intermediates such as oxaloactetate, thereby producing energy equivalents in the form of ATP. Glucogenic amino acids levels are known to decrease right after the transition from light to dark incubation in wild-type *Synechocystis* [20], alongside a substantial upregulation in genes involved in ATP synthesis [53]. This transition from light to dark is also accompanied by an increase in flux through the oxidative part of the pentose phosphate pathway (OPPP). OPPP is the major pathway of glucose catabolism in heterotrophic and mixotrophic cultures of *Synechocystis* [54] and is known to be used in conjunction with the Calvin cycle to regulate carbon fixation in autotrophic conditions [54].

The initiation of photosynthesis after the transition from light to dark uses energy compounds such as NADPH, ATP, and Calvin cycle intermediates. As these intermediates are shared with glycolysis, it is expected that respiration during dark time periods be closely linked to the initiation of photosynthesis in diurnally-growing *Synechocystis* [56]. Glycogen is the primary respiratory substrate in *Synechocystis* and its degradation is initiated by glycogen phosphorylase transferring orthophosphate to the non-reducing end of the glucose residue in glycogen and releasing glucose-1-phosphate, which feeds into glycolysis. Experiments with *Synechocystsis* mutants deficient in glycogen phosphorylase (ΔGlgP) found that the amount of dark respiration was 25% lower than that in the wild-type, following which the photosynthetic oxygen evolution rate reached its steady-state value at a later time [56], delineating a temporal dependence between glycolysis during the dark and photosynthesis during light. Our simulations also show accumulation of circulating Calvin cycle intermediates during the dark TPMs. Metabolites such as 3-phosphoglycerate, which is used to regenerate D-ribulose-1,5-bisphosphate (RuBP) during photosynthesis [57], and other glycolytic metabolites such as 2-phosphoglycerate and fructose-6-phosphate exhibit this phenomena. These metabolites are fed into glycolysis during the dark and enter the Calvin cycle during the transition from dark to light, suggesting that glycolytic intermediates produced during respiration in the dark are used for regenerating RuBP via the Calvin cycle during the induction of photosynthesis. Interestingly, the total accumulation of RuBP was higher in light than in the dark, implying preferential production and degradation of metabolites guided by the organism’s circadian clock. This is in alignment with cyanobacteria upregulating the oxidative pentose phosphate pathway in the absence of light [58].

Metabolite classes such as fatty acids, porphyrins, nucleotides, and proteins were selectively produced in the light as opposed to the dark TPMs, in agreement with literature. Fatty acid biosynthesis is known to increase with increasing light intensity in *Synechocystis* [59] and the responsible enzymes involved show increased synthesis during the light in *Cyanothece* as well [60]. Furthermore, photo-oxidative stress during photosynthesis gives rise to reactive oxygen species and initiates redox signaling. Ansong et al. [61] showed that several proteins involved in fatty acid biosynthesis are redox controlled in *Synechococcus elongatus* 7002, including acetyl CoA-carboxylase, which catalyzes the first step of fatty acid biosynthesis. Nucleotide and protein metabolism enzymes were shown to have the highest representation among all redox sensitive proteins, both of which show higher metabolite accumulation in light as opposed to dark in CycleSyn (Fig 2). Increased protein accumulation during light is also supported by an upregulation in the corresponding genes involved in protein synthesis in *Synechocystis* [38]. The increase in porphyrins such as heme during the light TPMs is the consequence of the increase in pigment production. Heme and chlorophyll are both tetrapyrrole pigments and hence share a common biosynthetic pathway [62]. Both chlorophyll and heme production is known to be upregulated during light in cyanobacteria [38,60], due to their central role in photosynthesis [63].

**Fig 3:**
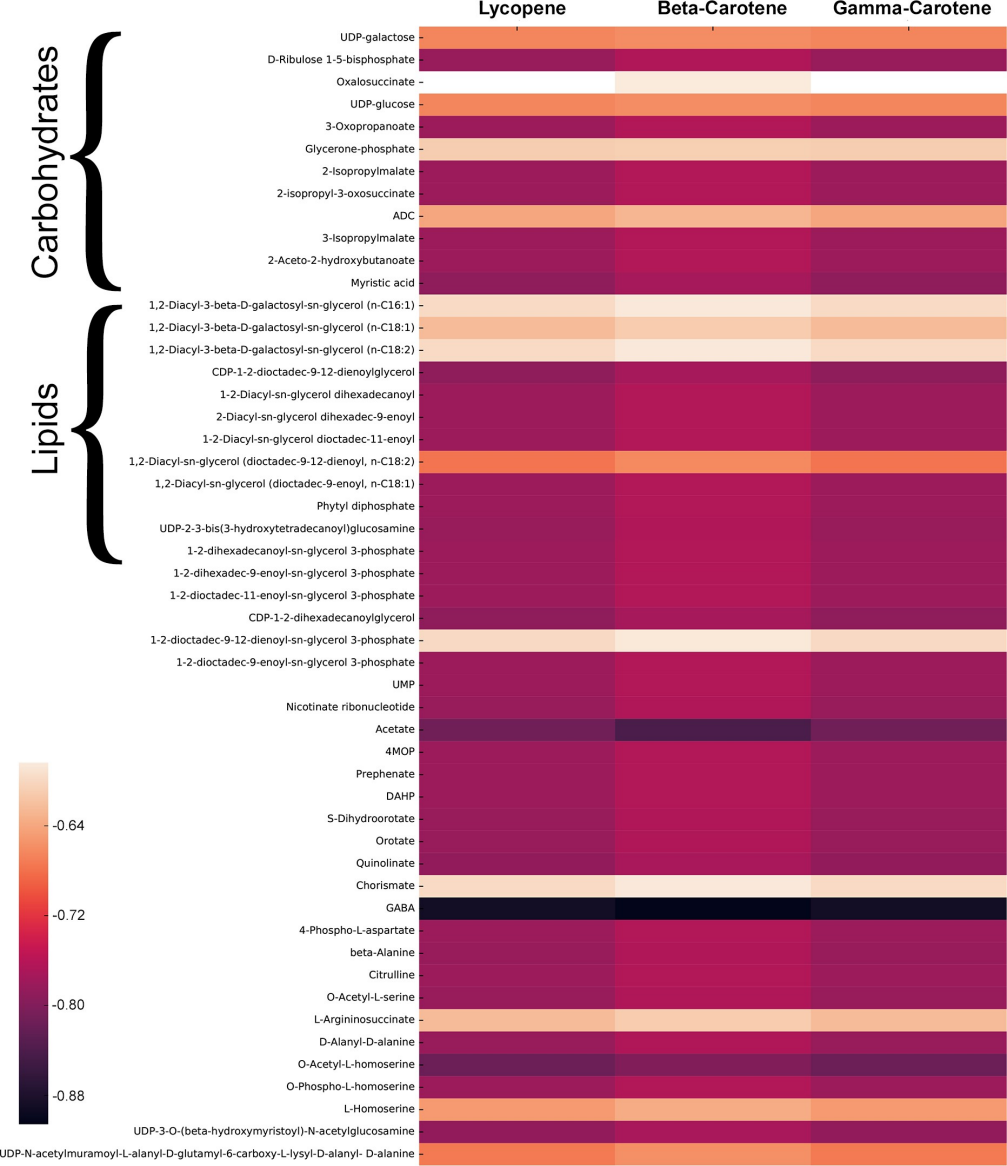
Metabolite-metabolite correlation analysis of the terpenes lycopene, beta-carotene and gamma-carotene. The maximum transfer flux for each metabolite obtained from FVA [49] was normalized with respect to the largest value recorded across all TPMs. Then, the non-parametric Spearman test with a p-value cut-off of 0.05 was used to find pairwise correlations of metabolites in the class terpenes with all other metabolites. Metabolite correlations with p-values of more than 0.05 have not been included and hence are left blank (depicted using white) in the figure.

Interestingly, terpenes such as lycopene and gamma-carotene are synthesized in the latter half of the day in CycleSyn, rising just before the transition to dark and maintaining these levels throughout the dark TPMs. We sought to investigate the source of this production using Metabolite-metabolite correlation analysis (MMCA) [64–67] (see Fig 3), which assesses metabolite concentration interdependencies using similarity metrics. These dependencies have been used before to explore the temporal organization of metabolic networks [68,69]. The analysis herein calculates pair-wise correlation coefficients between time-resolved transfer flux profiles for all metabolites using the non-parametric Spearman Test, employing a two-tailed test for hypothesis testing with a p-value cut-off of 0.05. The transfer flux for every metabolite was normalized with respect to the maximum value recorded across all TPMs and used as a proxy for metabolite concentrations. MMCA is usually employed on experimental datasets of metabolite concentrations, but CycleSyn’s ability to predict metabolite accumulation levels under a FBA paradigm enables us to use MMCA to study possible correlations between metabolite transfer fluxes. MMCA showed that metabolites of the pentose phosphate pathway such as Ribulose-1,5-bisphosphate (RuBP) and dihydroxyacetone phosphate (DHAP) are negatively correlated with terpenes such as gamma-carotene, beta-carotene, and lycopene, the three terpenes showing maximum production during late light (Fig 3), thus indicating that a rise in the levels of terpenes is associated with a fall in the levels of RuBP and DHAP. A similar phenomenon has been observed earlier by Ershov et al. [70] for a phototrophically growing *Synechocystis*, where terpenoid biosynthesis was stimulated by the addition of DHAP and other compounds of the pentose phosphate pathway, such as glyceraldehyde-3-phosphate (G3P) and D-ribulose 5-phosphate. Isoprenoid synthesis in *Synechocystis* occurs via the 2-C-methyl-D-erythritol pathway (MEP pathway), which starts with the condensation of glyceraldehyde 3- phosphate (GA3P) and pyruvate [71]. Thus, the substrates for terpenoid production are obtained from the metabolite products of photosynthesis such as DHAP and G3P. This also explains the temporal order of terpenoid production, wherein products of photosynthesis need to accumulate in order for the MEP pathway to be active, thus leading to terpene production in the late light, as replicated in CycleSyn. Furthermore, genes associated with the MEP pathway were seen to be upregulated in the dark in a diurnally cultured *Synechocystis*, such as those corresponding to phytoene dehydrogenase, phytofluene dehydrogenase, and the reaction CTP:2-C-Methyl-D-erythritol 4-phosphate cytidylyltransferase [38], which catalyzes the conversion of MEP into 4-(cytidine 5’-diphospho)-2-C-methyl-D-erythritol and constitutes the second step of the MEP pathway.

The metabolite class made up of RNA was found to be largely insensitive to the diurnal changes in metabolism, transfer fluxes ranging between 4.345 and 4.328 mmol gDW^-1^ hr^-1^. The time invariant nature of RNA production is consistent with a study that determined the total amount of tRNAs is relatively constant over a diurnal cycle in *Synechocystsis*, with the major RNA variability originating from long transcripts such as 16S rRNA and 23S rRNA [72] which are not captured in the present metabolic reconstruction.

### Glycogen accumulation

**Fig 4:**
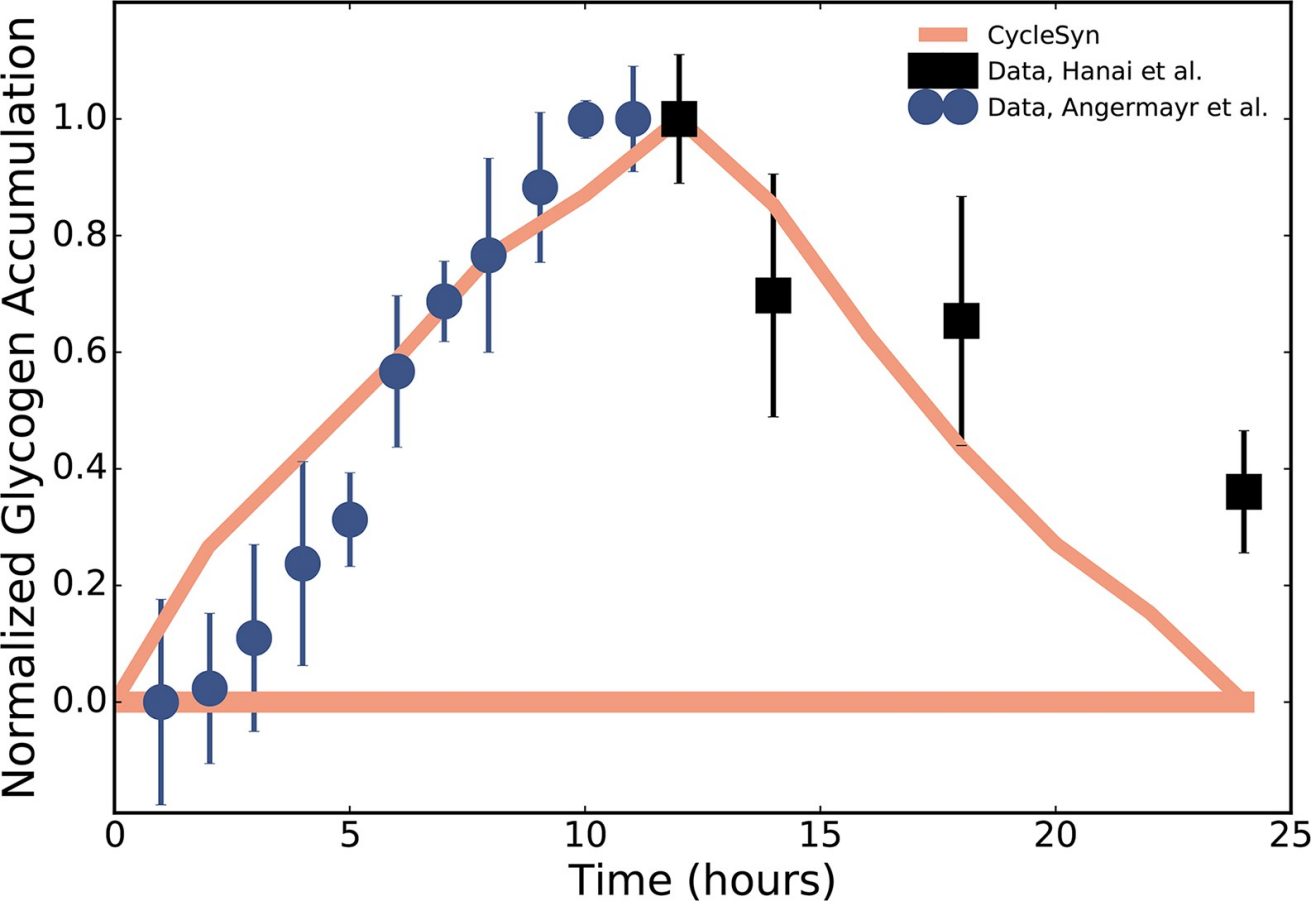
A comparison of experimentally observed increase in glycogen levels with CycleSyn predicted glycogen levels in a diurnally cultured *Synechocystis* across a 12h light/12h dark cycle. Normalized glycogen accumulation in a diurnally-growing *Synechocystis*. CycleSyn predictions (solid line, peach) are compared against experimentally measured glycogen content from Angermayr et al. [41] (solid blue circles) and Hanai et al. [20] (solid black squares). The glycogen levels for both the model predictions and the data have been normalized with respect to the highest value recorded throughout the light/dark cycle for uniformity and easier comparison.

Carbon fixation in *Synechocystis* during light exceeds needs (luxury uptake [73,74]) so as to catabolize those reserves in the dark to support growth and cellular maintenance. Glycogen is employed as such a reserve in *Synechocystis* [75,76], providing maintenance energy for cellular functions during dark periods [77]. As seen in Fig 4 and S1 Table, glycogen accumulates during the day and is gradually consumed during the night in CycleSyn. The highest glycogen transfer flux was recorded at the transition between light and dark in our simulations. Fig 4 contrasts the experimentally observed glycogen concentrations with the simulated glycogen accumulation levels. This comparison enables us to approximate the amount of a metabolite shuttled across TPMs after all its production/consumption reactions have taken place, so as to examine its overall temporal dynamics and contrast it with experimental values. Model-predicted glycogen dynamics is in accordance with experimental data, with the total glycogen content increasing gradually during light and reaching its peak level just before the onset of dark. The dark TPMs see a progressive decrease in glycogen as it is utilized as a respiratory substrate [77]. In particular, CycleSyn glycogen accumulation during light matches with the experimental levels seen by Angermayr et al. [41], but unlike Angermayr et al. we do not see a biphasic decline during late dark, which is also consistent with earlier studies [20,38,78]. Angermayr et al. [41] attribute the rapid decline in glycogen during the last two hours of dark to increased acetate accumulation and an upregulation of genes encoding the bidirectional NiFe-hydrogenase that is thought to help maintain the redox balance by reducing H^+^ [79]. CycleSyn did not predict a higher flux through NiFe-hydrogenase during the dark. In order to further ascertain the veracity of the model-predicted glycogen levels, we also compared the glycogen accumulation in the dark TPMs to data from Hanai et al. [20] (Fig 4). In this study *Synechocystis* was cultured under a 12hour light/12hour dark cycle and the glycogen concentration (as nmol per gm fresh weight (FW)) was measured at 0, 2, 6, and 12 hours after the transition to dark (Fig 4). As seen in Fig 4, CycleSyn predicted glycogen dynamics during the dark matches that seen by Hanai et al. [20].

Although CycleSyn predicts glycogen accumulation during light and degradation during the dark, the minimum possible transfer flux for glycogen is zero, indicating that other metabolites can serve as additional storage reserves. Isocitrate is found to be such a possible storage metabolite that is accumulated during light and degraded in the dark TPMs. Its catabolism occurs via isocitrate dehydrogenase, encoded by the *icd* (slr1289) gene which has been found to be upregulated in the dark as compared to light in a diurnally cultured *Synechocystis* by Saha et al. [38]. Experiments with *Synechocystis* sp. PCC 6803 impaired in glycogen synthesis have displayed overflow of pyruvate and 2-oxoglutarate [80,81], suggesting that carbon excess is directed preferentially into these compounds in the absence of glycogen. Isocitrate dehydrogenase catabolizes isocitrate to produce 2-oxoglutarate, whose central role in *Synechocystis* metabolism is discussed below and elucidated through metabolic control analysis.

### CycleSyn biomass production

**Fig 5:**
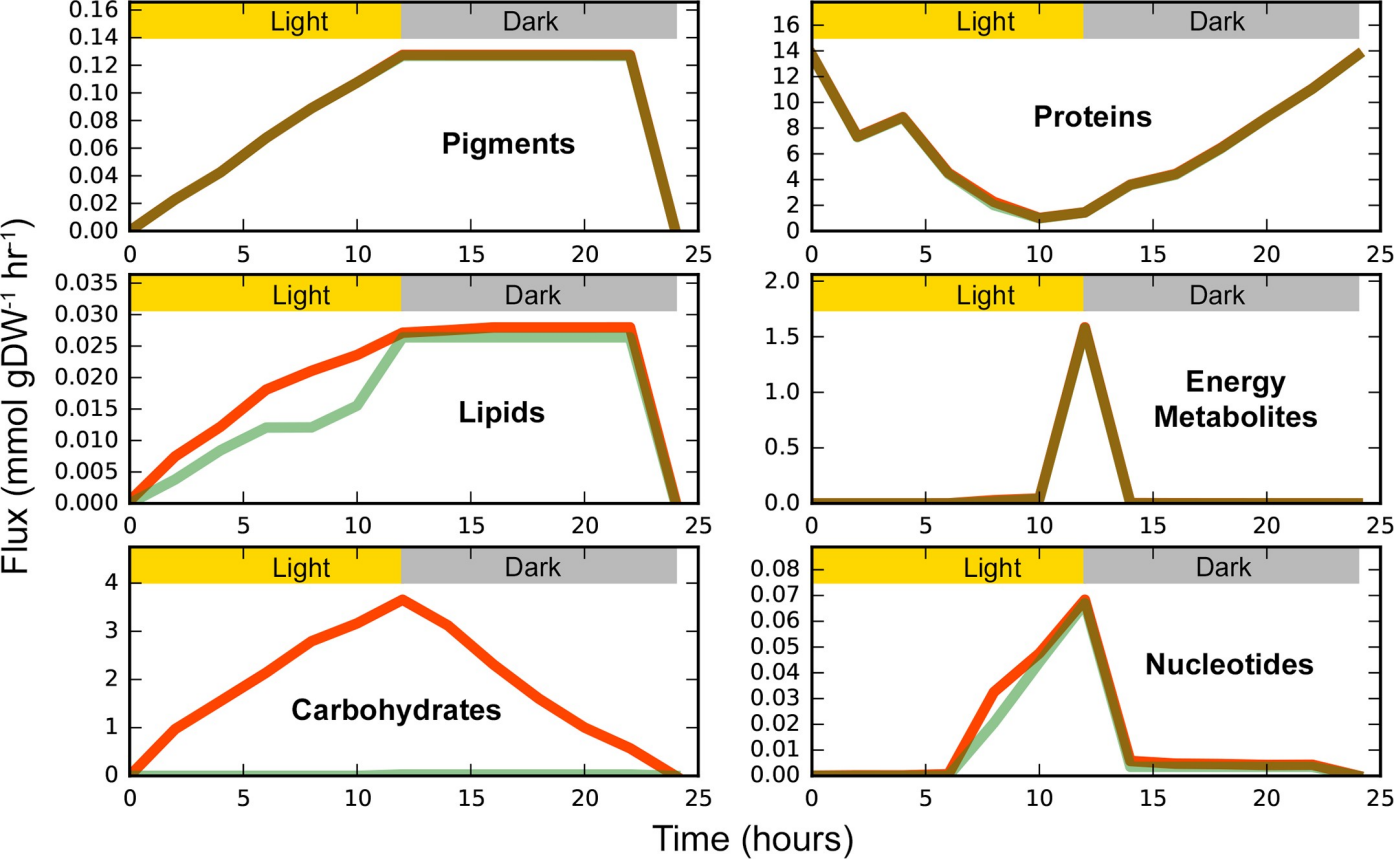
Category-wise temporal flux variations for biomass precursors over a 24-hour diurnal cycle. Maximum (red) and minimum (green) transfer fluxes for all metabolites that feed into the biomass equation (see supplementary S1 Table for the list of all biomass precursors and their transfer flux values) across all TPMs. The flux values are reported in units of mmol gDW^-1^ hr^-1^. Flux variability analysis (FVA) [49] was used to estimate individual reaction fluxes, by alternatively maximizing and minimizing flux through every reaction. The total flux for a category of transferring metabolites was determined by summing over the individual metabolite contributions. Metabolite annotations were taken from KEGG and provided in supplementary S6 Table.

The biomass equation approximates the composition of dry biomass and is used to drain biomass precursors in their physiologically relevant ratios. Fig 5 describes the sum of the maximum and minimum transfer fluxes for classes of biomass precursors over a 24-hour diurnal cycle (see S1 Table for the list of transfer fluxes of all biomass precursors). As seen in Fig 5 and S1 Table, biomass precursors such as carbohydrates and nucleotides are primarily produced during the day and sequestered in the night during the last TPM. Chlorophyll is synthesized during light as is known to occur in cyanobacteria [41], with these levels remaining constant during the dark TPMs.

By constraining reaction flux using transcriptomic data, we identified 110 reactions (see S2 Table) with active bounds, i.e. reactions whose flux constraints limit metabolism when the biomass objective function is maximized. This also resulted in a decrease in the biomass flux, which dropped by 10% (compared to the unconstrained flux case) corresponding to a doubling time of ~25 hours. As transcriptomic constraints induced a decrease in the biomass production flux, alleviating some or all of these bounds might allow for an increase in the maximal biomass produced.

The 110 reactions with active bounds tend to maintain active bounds in multiple TPMs. After adjusting for this reoccurence, 33 unique reactions were identified—one exclusively in dark TPMs and the rest during light TPMs. The reactions with active upper bounds belong mainly to central carbon and amino-acid metabolism, alongside peripheral metabolic pathways such as purine, pyrimidine, aminosugars, and lipid metabolism, and can be broadly linked to pyruvate and 2-oxoglutarate metabolism. Reactions involved in central carbon metabolism such as glycolysis and the pentose phosphate pathway also have active constraints, such as the conversion of 3-phosphoglycerate to 1,3-bisphospho-D-glycerate and the reaction between ribose-5-phosphate and D-xylulose5-phosphate to produce glyceraldehyde-3-phosphate and sedoheptulose-7-phosphate. Furthermore, reactions belonging to glucogenic amino acid metabolism are also found to have active reaction bounds. Glucogenic amino acids such as lysine and aspartate yield through catabolism pyruvate or TCA cycle metabolites. As the TCA cycle is the primary source of ATP in cyanobacteria, upregulating these reactions during early light would allow for a greater TCA cycle turnover, leading to more biomass production and hence enhanced growth. The need to increase 2-oxoglutarate production and thus TCA cycle turnover is further evidenced by the constriction of reactions such as L-Phenylalanine:2-oxoglutarate aminotransferase, L-Aspartate:2-oxoglutarate aminotransferase, L-Valine:2-oxoglutarate aminotransferase, and L-Leucine:2-oxoglutarate aminotransferase in the direction of 2-oxoglutarate production. Enhanced pyruvate production has led to greater biomass production in cyanobacteria in an earlier study [27]. Modifying glycolytic pathways and the Calvin Benson cycle in *Synechococcus elongatus* PCC 7942 to redirect flux towards carbon fixation increased the intracellular pool of pyruvate, which led to about a three-fold increase in growth under both light and dark conditions [27].

In order to further investigate the central roles played by pyruvate and 2-oxoglutarate, we used metabolic control analysis [82] to identify reactions that most affect the biomass production upon a perturbation in their corresponding enzyme levels. A 1% enzyme perturbation was considered and the flux control coefficient (FCC) calculated for every reaction using transcript levels as proxies for the enzyme levels [83] (see Materials and Methods). FCCs provide a quantitative measure of the degree of control a particular enzyme exerts on the reaction flux of interest. Interestingly, of all the reactions considered, only two affected the final biomass production flux and only during light TPMs. These included reactions L-Tyrosine:2-oxoglutarate aminotransferase and L-Phenylalanine:2-oxoglutarate aminotransferase with FCCs of 0.016 and 0.04, respectively. Both these reactions are controlled by the same set of isozymes in *Synechocystis* which are not shared by any other reaction and both bidirectional reactions operate selectively in the direction of 2-oxoglutarate synthesis, alluding to the importance of 2-oxoglutarate in *Synechocystis* growth. The intracellular concentration of 2-oxoglutarate in *Synechocystis* has been implicated in the regulation of the coordination of carbon and nitrogen metabolism [84]. As *Synechocystis* lacks the traditional 2-oxoglutarate dehydrogenase complex, 2-oxoglutarate acts as the final carbon skeleton for nitrogen. It is used to sense changes in the cell’s nitrogen status [85] and provides the carbon backbones needed for synthesizing amino acids such as glutamate, glutamine, proline, and arginine biosynthesis via the GS-GOGAT cycle [86].

### Addition of nitrogen fixation

**Fig 6:**
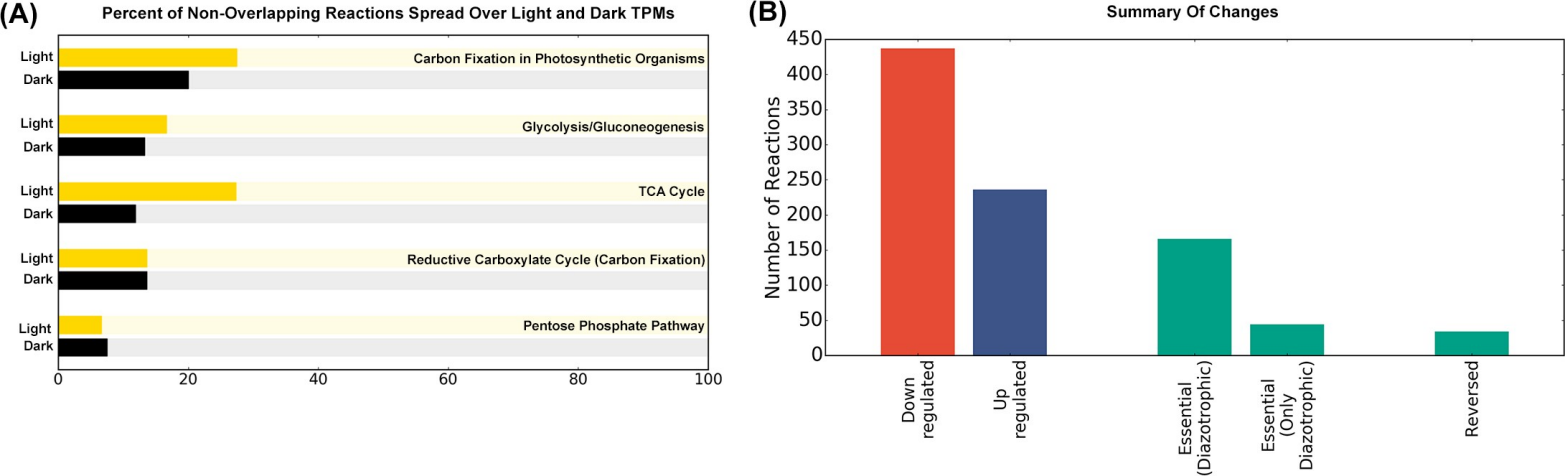
A quantitative summary of changes when diazotrophy was introduced in wild-type *Synechocystis* sp. PCC 6803. (A) The figure represents the percent of reactions with non-overlapping flux ranges across all light (yellow bars) and all dark (black bars) TPMs respectively. Pathway annotations from *i*Syn731 and KEGG were used. (B) The graph summarizes the metabolic changes detected upon introducing diazotrophy in *Synechocystis*, in terms of the number of reactions downregulated (red), upregulated (blue), essential, only under diazotrophy, and reversible reactions whose directions were switched in a diazotrophic *Synechocystis* as compared to the wild-type.

Nitrogen Fixation was introduced to the model by including the relevant reactions from *i*Cyt773, the GSM model for *Cyanothece* [28], and constraints to ensure that anoxic conditions are maintained during nitrogen fixation (see Materials and Methods), as the nitrogen-fixing enzyme nitrogenase is irreversibly inhibited by oxygen. Transcriptomic data from wild-type *Synechocystis* [38] was used to restrict reaction flux bounds, so as to identify the reactions that need to be regulated differently in order to fix nitrogen while maintaining growth. S3 Table lists the 672 reactions with flux ranges that change compared to the earlier-described diurnally growing wild-type *Synechocystis* (i.e. the feasible flux range associated with these reactions do not overlap with the flux range associated with the wild-type strain). 236 reactions (107 unique reactions after adjusting for TPM multiple participation) were upregulated and 436 reactions (225 unique) were downregulated upon the introduction of nitrogen fixation. A total of 166 reactions (128 unique) were found to be essential under diazotrophic conditions, i.e. had strictly positive or strictly negative flux profiles, out of which 44 reactions were non-essential under wild-type conditions. These reactions are indicators of the metabolic alterations required as a result of the introduction of diazotrophy in *Synechocystis* (see Fig 6 and S3 Table).

**Fig 7:**
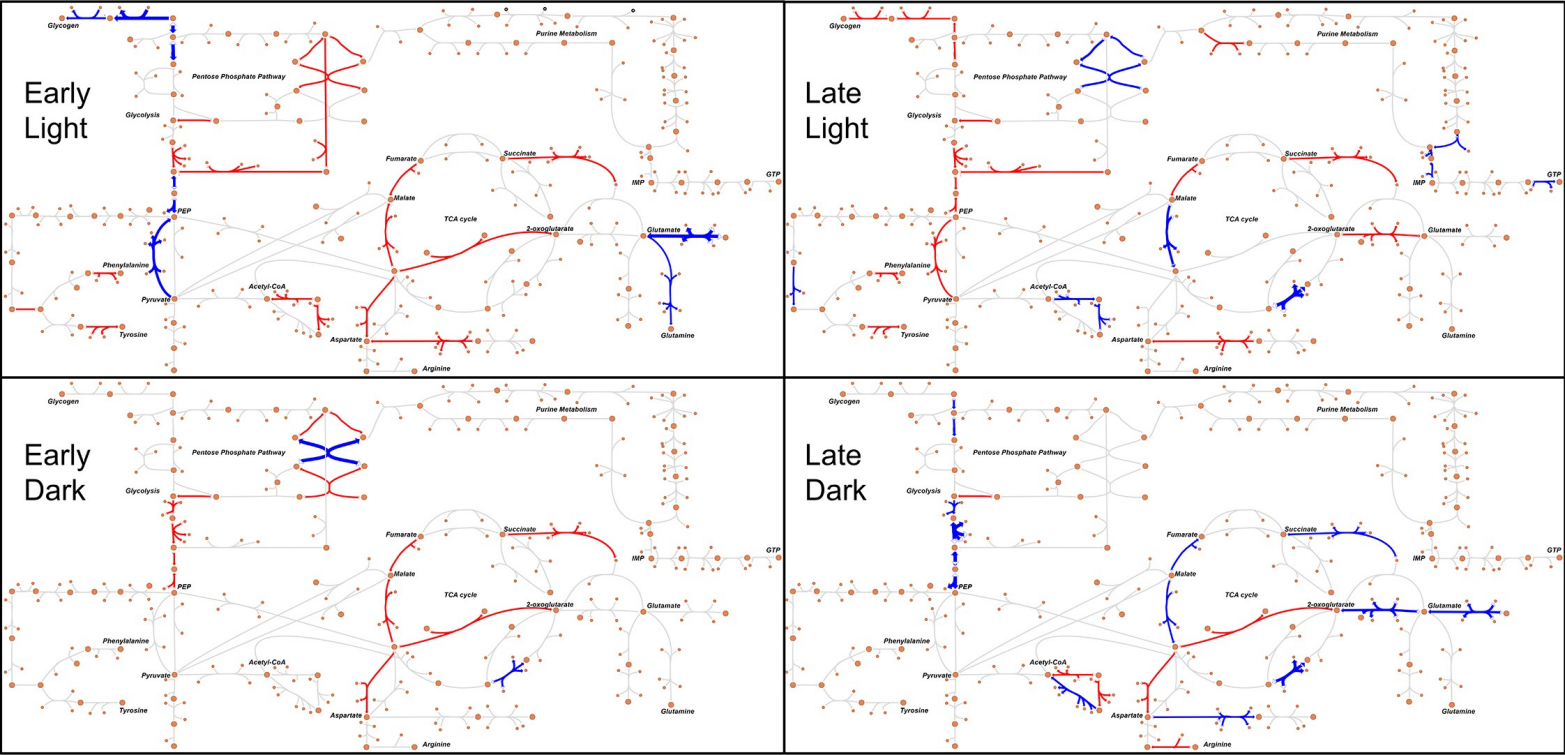
The effect of introducing diazotrophy in wild-type *Synechocystis* sp. PCC 6803. Visualized networks display the non-overlapping reactions that were upregulated (blue) and downregulated (red) under diazotrophic conditions in four TPMs representative of early light, late light, early dark, and late dark. The arrow widths are proportional to the amount of non-overlap between the reactions in the two conditions simulated. The web-tool Escher was used to construct the flux metabolic networks [89].

Reactions with a perturbed flux profile mainly belong to pathways of carbon and amino-acid metabolism, and target the two important modifications required for nitrogenase to function–increased ATP availability and an anaerobic environment. Upregulation of reactions such as L-Aspartic acid:oxygen oxidoreductase help maintain the latter, while the former is addressed by reactions belonging to glycolysis, TCA cycle, and photosynthesis in the light TPMs, i.e. TPMs 1 to 6, glycogen synthesis, and oxidative pentose phosphate pathway in the dark TPMs, i.e. TPMs 7 to 12 (Fig 7 and S3 Table). As nitrogen fixation is an energy-intensive process, requiring 16 ATP molecules and eight reducing equivalents for every molecule of dinitrogen fixed, this energy is being provided by the coordinated actions of these pathways, while the required reducing equivalents are being supplied by the upregulation of Ferredoxin:NADP+ oxidoreductase reaction, which converts NADP to NADPH while oxidizing ferredoxin. A similar phenomenon is seen in a diurnally cultured C*yanothece*, where transcriptomic analysis shows simultaneous upregulation of entire pathways involved in respiration and energy metabolism, such as pentose phosphate pathway, TCA cycle, glycolysis, and amino-acid metabolism in the dark [87]. *Cyanothece* emerges as a natural comparison for the hypothesized diazotrophic *Synechocystis* as in order to accommodate both nitrogen fixation and photosynthesis, *Cyanothece* temporally separates the two incompatible processes [88].

A number of reactions with highly changed flux ranges are similar to the temporal distribution of flux seen in the nitrogen-fixing *Cyanothece*. For instance, there is an increase in flux in the reactions responsible for glycogen degradation during late dark, which is fed into glycolysis via glucose-1-phosphate, so as to fuel the higher energy demands associated with nitrogen fixation. Similar to the diazotrophic *Cyanothece*, glycogen degradation in CycleSyn proceeds via glycolysis, the oxidative pentose phosphate pathway, and the TCA cycle so as to provide the cell with ATP, cellular precursors, and pyrimidine nucleotides. Increased respiration of carbohydrate reserves in the dark produces NADPH and succinate, which transfers electrons via NADPH dehydrogenase and succinate dehydrogenase into the plastoquinone pool [90], towards the terminal electron acceptor. This electron transport due to respiration sets up a proton gradient and drives ATP production. The increased flux towards succinate is evinced by the upregulation of (S)-Malate hydrolyase in the dark TPMs which reversibly converts malate into fumarate [47]. A similar upregulation is seen in *Cyanothece* BG 043511 as well, where an increase in nitrogen fixation at night coincided with a rise in respiratory electron transport [91]. Furthermore, as fixed nitrogen is incorporated via arginine and aspartate metabolic pathways, a number of reactions belonging to these pathways also show upregulation in the dark in a diazotrophic *Synechocystis* as opposed to the wild-type.

### Conclusion

Modelling phototrophic growth using constraint-based optimization techniques necessitates modeling contributions beyond conventional flux balance analysis. Since cyanobacteria show strong diurnal rhythms in its lifestyle, translating that phenotype into a metabolic model requires new approaches that enable us to incorporate and replicate those temporal metabolic reorganizations. Diurnal oscillations in Cyanobacteria have been the focus of many studies [6,17,53] but those have mainly been concentrated on the associated transcriptomic changes or a subset of its entire metabolism. In this work, we bridge that gap by developing a diurnal model of *Synechocystis* metabolism. We accommodated the cyanobacterial circadian clock and its influence on the underlying metabolic machinery by employing temporally-resolved transcriptomic data. The developed formalism was able to replicate the changes in metabolism observed in *Synechocystis* over a diel light-dark cycle. It should be noted here that CycleSyn does not assume that metabolite concentrations, enzyme activities, or reaction rates are governed solely by mRNA expression levels. It is well known that the true flux through a reaction depends on the enzyme kinetics and expression, alongside metabolite concentrations (Michaelis-Menten kinetics). The biological rationale behind CycleSyn is that expression data provides measurements of the level of mRNA available for each gene. If there was a limited accumulation of an enzyme in a particular TPM with respect to the others, the (relative) level of mRNA can be used as an approximate upper bound for the maximum protein available. This can then be used to constrict the maximum permissible flux through a reaction, effectively reshaping the metabolic flux cone. This enables a systematic extension of flux balance analysis by making use of temporal changes in expression levels to predict the metabolic capacity of *Synechocystis* over a 24-hour period. The correspondence between transcript levels, reaction fluxes and metabolite levels has been elucidated before [92] where accuracy in predicting the direction of change (increase/decrease) in metabolite levels increased by 90% when constraints derived from transcriptomic data were included in the metabolic model of a maize leaf.

The 24hr model provides a time-course for all reaction fluxes and metabolite levels. The model predictions are aligned well with several known phenomena in a diurnally-controlled cyanobacterial phototrophic metabolism. We predicted that glycogen was accumulated during light and degraded steadily during dark, as is seen in *Synechocystis* [38,41]. Different pathways were upregulated during the light and dark phases, highlighting the variations in metabolism occurring due to light availability. Glycolysis intermediates produced during respiration in the dark were being used for regenerating RuBP via the Calvin cycle during the induction of photosynthesis. Levels of amino acids produced from glycolytic precursors such as pyruvate and alpha-ketoglutarate decreased at the transition from light to dark incubation in wild-type *Synechocystis* alongside a substantial upregulation in genes involved in ATP synthesis, as is seen experimentally [20], [53]. CycleSyn also predicted pyruvate metabolism as a bottleneck in biomass synthesis. Redirecting flux towards pyruvate synthesis can increase carbon fixation and hence biomass formation, as is seen in *Synechococcus elongatus* PCC 7942 [27].

We also treated *Synechocystis* as an example cyanobacterium to predict the various metabolic pathways that would need to be regulated in a photosynthetic organism so as to incorporate the two inherently incompatible processes of photosynthesis and nitrogen fixation. The introduction of nitrogen fixation drew parallels from the non-heterocystous cyanobacteria *Cyanothece* ATCC 51142, which temporally separates the two antagonistic processes. In doing so it fixes glycogen during the day and uses it as a respiratory product in the dark, thus also achieving the anoxic conditions required for nitrogenase activity. This necessitates a reorganization of the cellular metabolic processes, with dominant flux-carrying pathways differing during the light and dark periods. The model predicted changes in pathways of carbon fixation and amino acid synthesis upon introduction of diazotrophy in *Synechocystis*. The dark phase in *Cyanothece* is known to have a high protein turnover, with upregulation of amino-acid biosynthesis pathways due to the increased nitrogen sequestration. Pathways such as arginine and aspartate metabolism were consequently upregulated in the dark, due to the need to sequester the fixed nitrogen. CycleSyn predicted the high-energy demands associated with nitrogen fixation to be met by increased flux through TCA cycle and the pentose phosphate pathway, maintained by higher glycogen synthesis and remobilization. Furthermore, oxygen scavenging reactions such as L-Aspartic acid:oxygen oxidoreductase were upregulated across dark TPMs, due to their oxygen-scavenging role which is required to maintain the anaerobic conditions required for nitrogenase to function.

The developed framework enables analyzing a time-variant GSM model while preserving the fundamental time-invariant assumption of conventional flux balance analysis. It improves upon existing techniques of diurnal simulations of metabolism while maintaining a linear programming problem resulting in low computational costs. The formulation can readily be customized to accommodate quantitative measurements of reaction fluxes over a 24-hour cycle. This will also constrain the feasible solution space and thereby improve the precision and accuracy of model predictions [93]. We expect this and similar methods to become instrumental in understanding, analyzing, and predicting temporal metabolic flux variations.

## Methods

### CycleSyn construction

We constructed CycleSyn using the *i*Syn731 genome-scale metabolic (GSM) model for *Synechocystis* sp. 6803 [28] as a scaffold. *i*Syn731 was updated to reflect the latest annotations made to the *Synechocystis* genome as present in CyanoBase (see supplementary S5 Table). Additions include the Entner-Doudoroff pathway, the phosphoketolase pathway, and the light-independent serine biosynthesis pathway [94–98], among others. Metabolite and reaction IDs were borrowed from ModelSEED [99] wherever possible. From 1,156 reactions and 1,003 metabolites, the model increased to 1,165 reactions and 1,008 metabolites. Flux variability analysis was performed on this model to ensure that it is free of any thermodynamically infeasible loops which can carry unbounded flux.

The 24-hour model consists of 12 individual Time Point Models (TPMs), each approximating reaction fluxes over a 2-hour period. The first TPM covers the first two hours of the light period (L0-L2), the second TPM covering the next two hours of light (L2-L4), with the pattern continuing until TPM12 which contains the last two hours in the dark period (D10-L0). Each time-point model was made by duplicating reactions (by appending ‘_tpmX’ to reaction name, where X is the TPM number ranging from 1 to 12) and metabolites (denoted by appending ‘[tpmX]’ to the metabolite name) in the base model.

A single biomass reaction occurs in TPM12 to account for organism growth. All metabolites except photons and protons that are present in the cytosol and carboxysome are transferred unidirectionally from the *n* to the *n*+1 TPM using transfer reactions. A transfer reaction *j* for a metabolite *i* in a TPM *k* always operates in the forward direction from TPM *k* to TPM *k*+1 such that

$${metabolite}_{i,k}\overset{{transfer}_{j,k}}{\to }{metabolite}_{i,k+1}$$

Photosynthesis is only allowed to occur in a TPM *k* if it contains the necessary chlorophyll. The chlorophyll balance on a TPM equates the difference between total chlorophyll synthesis and degradation fluxes to the difference between the chlorophyll transfer fluxes that exit and enter the TPM. This is mathematically represented as:

$$\sum v_{chlorophyll\;synthesis,k}-\sum v_{chlorophyll\;degradation,k}=v_{pigment\;transfer,\;k\;to\;k+1}-v_{Pigment\;Transfer,\;k-1\;to\;k}$$

Where *v*_*Pigment Transfer*, *k to k*+1_ refers to the flux through the chlorophyll transfer reaction from TPM *k* to TPM *k*+1.

The chlorophyll transfer flux from a TPM *k* to TPM *k*+1 represents the cumulative chlorophyll accumulation (i.e., from TPM 1 to TPM *k*). We approximate this as a linearly increasing function with respect to time for a single TPM. Hence, the average amount of chlorophyll made in a TPM *k* can now be calculated as

$$<v_{chlorophyll,k}>=\frac{1}{2}(v_{Pigment\;Transfer,\;k\;to\;k+1}-v_{pigment\;transfer,\;k-1\;to\;k})$$

The constraint levied on the model that couples photosynthesis to chlorophyll availability is expressed as:

$$v_{Photosynthesis,\;k}\leq \frac{1}{2}(v_{Pigment\;Transfer,\;k\;to\;k+1}-v_{pigment\;transfer,\;k-1\;to\;k})M_{C}$$ (1)

where *M*_*C*_ is a constant large enough not to constrain flux through photosynthesis reactions and *k* the Time Point Model. *v*_*Pigment Transfer*, *k to k*+1_ refers to the flux through the chlorophyll transfer reaction from TPM *k* to TPM *k*+1. This implies that amount of chlorophyll available to carry out photosynthesis is equal to the difference between the amount transferred in to time point *k* and the amount transported out to point *k*+1, divided by two. A value of 1000 for *M*_*C*_ predicts photosynthetic oxygen evolution (estimated by the output flux through the oxygen exchange reaction during the light TPMs) ranging between 173 to 169 *mmol O*_2_ (*gm chlorophyll*)^−1^ which is in the same order as that of wild-type *Synechocystis*. A wide range of values have been reported experimentally ranging from 225 *mmol O*_2_ (*gm chlorophyll*)^−1^ (*hr*)^−1^ [100] to 380 *mmol O*_2_ (*gm chlorophyll*)^−1^ (*hr*)^−1^ [101], precluding the matching of a single value. The set of photosynthetic reactions included in this constraint are cytochrome b6/f complex, cytochrome c oxidase cytochrome oxidase bd, Mehler reaction, photosystem I (plastocyanin), photosystem I (ferrocytochrome), photosystem II, and succinate dehydrogenase (periplasm) (see S4 Table for model reaction identifiers and reaction descriptions for reactions belonging to this constrained set).

The optimization formulation used to determine maximum biomass production flux is

$$maximize\;v_{biomass,\;TPM12}$$ (2)

subject to

$$\sum_{j\in J}S_{ijk}v_{jk}=0,\;\forall i\in I,k\in K$$ (3)

$$v_{CO_{2}\;uptake,k}\leq 1.1,\;k=1,\ldots ,6$$ (4)

$$v_{Photon\;uptake,k}\leq 60,\;k=1,\ldots ,6$$ (5)

$$v_{ATP\;maintenance,k}\geq 10,\;\forall k\in K$$ (6)

$$v_{jk}^{LB}\leq v_{jk}\leq v_{jk}^{UB},\;\forall j\in J,k\in K$$ (7)

$$0\leq v_{j,k}\leq 10,000,\;\forall j\in J^{Transfer},k\in K$$ (8)

$$v_{Photosynthesis,k}\leq \frac{1}{2}(v_{Pigment\;Transfer,\;k\;to\;k+1}-v_{pigment\;transfer,\;k-1\;to\;k})M_{C},\forall k\in K$$ (9)

where *S*_*ijk*_ is the stoichiometric coefficient for metabolite *i* in reaction *j* and TPM *k*, $v_{jk}^{LB}$ and $v_{jk}^{UB}$ are the upper and lower flux bounds for reaction *j* in TPM *k*.*I*,*J*, and *K* denote the sets of total metabolites, reactions, and Time Point Models (TPMs), respectively, and *J*^*Transfer*^ the set of all transfer reactions. Constraints (4) and (5) refer to carbon (as CO_2_) and photons being supplied to only the light TPMs. The maximum amount of photons supplied to a TPM are such that it is not growth-limiting but at the same time not in excess so as to not trigger light-sensitive reactions. $v_{CO_{2}\;uptake,k}$ and *v*_*Phonton uptake*,*k*_ represent the carbon and photon uptake reactions, and *v*_*ATP maintenance*,*k*_ is the ATP maintenance requirement for a TPM *k*. Every transfer reaction j in TPM *k* is constrained to have a non-negative flux by Eq (8).

As all the individual metabolites are transferred (only forward) throughout all TPMs, they may give rise to cycles that can carry unbounded flux. Hence, to prevent this cycling, the sum of transfer fluxes was set to a scalar *f* that was identified by solving a modified pFBA formulation.

$$Minimize\;f=\sum_{k\in K}\sum_{j\in J^{Transfer}}v_{jk}$$ (10)

subject to

$$\sum_{j\in J}S_{ijk}v_{jk}=0,\;\forall i\in I,k\in K$$

$$v_{CO_{2}\;uptake,k}\leq 1.1,\;k=1,\ldots ,6$$

$$v_{Photon\;uptake,k}\leq 60,\;k=1,\ldots ,6$$

$$v_{ATP\;maintenance,k}\geq 10,\;\forall k\in K$$

$$v_{jk}^{LB}\leq v_{jk}\leq v_{jk}^{UB},\;\forall j\in J,k\in K$$

$$0\leq v_{j,k}\leq 10,000,\;\forall j\in J^{Transfer},k\in K$$

$$v_{Photosynthesis,k}\leq \frac{1}{2}(v_{Pigment\;Transfer,\;k\;to\;k+1}-v_{pigment\;transfer,\;k-1\;to\;k})M_{C},\;\forall k\in K$$

$$v_{Biomass,TPM12}=v_{Biomass,TPM12}^{max}$$ (11)

where *J*^*Transfer*^ is the set of all transfer reactions and $v_{Biomass,TPM12}^{max}$ the maximum biomass production flux as determined by (2).

In photosynthetic organisms such as *Synechocystis*, a proton gradient is generated during photosynthesis across the thylakoid membrane that drives ATP formation. Transferring this gradient across time points would result in an untenable way for storing energy outside of storage compounds. To this end, protons and photons were not transferred across TPMs. The following optimization model formulation is used to carry out flux variability analysis (FVA):

$$Maximize/Minimize\;v_{jk}$$ (12)

subject to

$$\sum_{j\in J}S_{ijk}v_{jk}=0,\;\forall i\in I,k\in K$$

$$v_{CO_{2}\;uptake,k}\leq 1.1,\;k=1,..,6$$

$$v_{Photon\;uptake,k}\leq 60,\;k=1,..,6$$

$$v_{ATP\;maintenance,k}\geq 10,\;\forall k\in K$$

$$v_{jk}^{LB}\leq v_{jk}\leq v_{jk}^{UB},\;\forall j\in J,k\in K$$

$$v_{Photosynthesis,k}\leq \frac{1}{2}(v_{Pigment\;Transfer,\;k\;to\;k+1}-v_{pigment\;transfer,\;k-1\;to\;k})M_{C},\forall k\in K$$

$$0\leq v_{j,k}\leq 10,000,\;\forall j\in J^{Transfer},k\in K$$

$$v_{Biomass,TPM12}=v_{Biomass,TPM12}^{max}$$

$$\sum_{k\in K}\sum_{j\in J^{Transfer}}v_{jk}\leq f$$ (13)

CPLEX solver (version 12.1, IBM ILOG) was used in the GAMS (version 23.3.3, GAMS Development Corporation) environment for solving all optimization models. All computations were carried out on dual 10-core and 12-core Intel Xeon E5-2680 and Intel Xeon E7-4830 quad 10-core processors that are the part of the ACI cluster of High Performance Computing Group of The Pennsylvania State University. Numerical scaling issues were not observed when solving CycleSyn.

#### Incorporating nitrogen fixation into *Synechocystis*

Nitrogen fixation was included in the model with the addition of the nitrogen fixation reaction from *i*Cyt773 [28] (i.e. reduced ferredoxin:dinitrogen oxidoreductase (ATP-hydrolyzing)) along with the diffusion transport reactions (i.e., nitrogen exchange with the environment, nitrogen transport between the extracellular compartment and the periplasm, and between the periplasm and the cytosol) required for nitrogen to enter and leave the cell.

As oxygen irreversibly inhibits nitrogenase, an anaerobic environment is required for successful nitrogen fixation. *Cyanothece* achieves this by utilizing glycogen in the early dark period as a carbon source, thereby also consuming oxygen [102]. The following equations represent this inhibition—if the oxygen transporter is carrying flux out of a TPM (i.e. if oxygen is still present within the cell and has not been consumed during the 2-hour period), then nitrogen fixation cannot occur.

$$LB_{N_{2}fixation,k}y_{N_{2}fixation,k}\leq v_{N_{2}fixation,k}\leq UB_{N_{2}fixation,k}y_{N_{2}fixation,k}$$ (14)

$$LB_{O_{2}\;export,k\;to\;k+1}y_{O_{2}\;export,k\;to\;k+1}\leq v_{O_{2}\;export,k\;to\;k+1}\leq UB_{O_{2}\;export,k\;to\;k+1}y_{O_{2}\;export,k\;to\;k+1}$$ (15)

$$y_{N_{2}fixation,k}+y_{O_{2}\;export,k\;to\;k+1}\leq 1$$ (16)

Where *y* is a binary variable that is 1 when the corresponding reaction is active and 0 otherwise.

Given the handful of binaries employed, solve time for a single FBA simulation with the above constraints incorporated is ~3 seconds, which is almost equal to the solve time of the LP.

#### Transcriptional constraints

Transcriptomic data [38] was used to further constrain reaction fluxes using the E-flux approach [39], as gene expression data can provide insights into the temporal variations in *Synechocystis* metabolism over a light-dark cycle. Gene-Protein-Reaction (GPR) relationships are evaluated for each reaction and the upper and lower bounds determined by flux variability analysis of the corresponding reactions are reduced by a factor corresponding to the expression ratio [39]. Flux bounds from the original *i*Syn731 model (*i*.*e*., not segmented into TPMs) were used, so that the metabolic capacity of every TPM is equivalent before introducing omics-derived flux constraints. Expression ratios of normalized gene intensities were determined for every time point as the expression level at a time point divided by the maximum expression of the gene across all time points, and not scaled using a non-linear transform such as the sigmoid scale. For reactions controlled by isozymes, the largest ratio among all contributing isozymes was used, as when multiple enzymes have the same enzymatic activity the one with the largest amount governs flux through the reaction. In the case of protein subunits the smallest ratio was used, as flux through the reaction is limited by the amount of the subunit component present in the lowest concentration. This is incorporated into the optimization framework presented earlier by modifying Eq (7) as

$$v_{jk}^{LB}\leq v_{jk}\leq v_{jk}^{UB}a_{jk},\;\forall j\in J,k\in K$$

Where *a*_*jk*_ is the expression ratio corresponding to reaction *j* in TPM *k*.

Out of the 449 reactions for whom transcriptomic constraints were applied, 55 reactions were controlled by isozymes/protein subunits. Although the E-flux method reduces the feasible solution space by restricting the upper bound of reactions, the presence of transfer reactions from one TPM to the next permits the same reaction to occur in another TPM if restricted in the current. Therefore, CycleSyn is not likely to yield an infeasible LP.

Transcriptional constraints were added for both the diazotrophic and non-diazotrophic models to glean information about the pathways and specific reactions that would need added regulation for *Synechocystis* to produce maximal biomass while fixing nitrogen.

#### Calculation of flux control coefficients

In our model, we determined the impact on the final biomass production flux brought about by a perturbation in the individual enzyme levels taken one at a time, taking mRNA expression to be a proxy for enzyme levels. Transcriptomic bounds constraining reactions in CycleSyn were perturbed by 1% (= Δ*x*_*ase*_) and the corresponding change in biomass flux recorded (= Δ*v*_*biomass*_) and reported using flux control coefficients defined as:

$$C_{x_{ase}}^{v_{biomass}}=\frac{\Delta v_{biomass}}{\Delta x_{ase}}.\frac{x_{ase}}{v_{biomass}}$$

#### Metabolite-metabolite correlation analysis (MMCA)

MMCA determines the interdependence between metabolite concentrations by calculating pair-wise correlation coefficients between metabolite pairs. Here we used the non-parametric Spearman test [103] to calculate correlation coefficients between the transfer flux profiles of metabolites as predicted by CycleSyn. A two-sided test with a p-value cut-off value of 0.05 was used for hypothesis testing. The scientific python module (scipy) in Python 2.7 was used to perform all calculations. If there are n pairs of observations in two continuous distributions, and *u*_*i*_ is the rank of the *i*^*th*^ observation in the first sample and *v*_*i*_ is the rank of the *i*^*th*^ observation in the second sample, Spearman’s rank correlation coefficient (= *r*_*s*_) is given as

$$r_{s}=\frac{n\sum_{i=1}^{n}u_{i}v_{i}-(\sum_{i=1}^{n}u_{i})(\sum_{i=1}^{n}v_{i})}{\sqrt{\left[n\sum_{i=1}^{n}u_{i}^{2}-{\left(\sum_{i=1}^{n}u_{i}\right)}^{2}][n\sum_{i=1}^{n}v_{i}^{2}-{\left(\sum_{i=1}^{n}v_{i}\right)}^{2}\right]}}$$

## Supporting information

S1 Table Transfer fluxes (in mmol per gDW hr) of all biomass precursors, over all time points for wild-type *Synechocystis*. (XLSX)

S2 Table List of reactions with active constraints in wild-type *Synechocystis*. (XLSX)

S3 Table List of reactions from mutant (nitrogen-fixing) *Synechocystis* with non-overlapping flux ranges as compared to wild-type *Synechocystis*. (XLSX)

S4 Table List of reactions whose flux was constrained in a TPM by the amount of pigment being produced in that TPM. (XLSX)

S5 Table The updated *i*Syn731 genome-scale model. (XLSX)

S6 Table List of metabolite annotations used in Fig 2 and Fig 4 with metabolite transfer fluxes across all TPMs. (XLSX)

S7 Table Fluxes of all nutrient exchanges present in CycleSyn. (XLSX)

S1 Fig Distribution of total number (black bars) and transcriptionally constrained reactions (grey bars) across metabolic pathways in the updated *i*Syn731 genome-scale model. (PNG)

S1 File Escher map used to generate metabolic flux maps in Fig 7 (JSON file). (JSON)

S2 File CycleSyn model in SBML format. (ZIP)
