# Supplementary figures and images for: A diurnal flux balance model of Synechocystis sp. PCC 6803 metabolism
Source: PLoS Comput Biol. 2019 Jan 24;15(1):e1006692. doi: 10.1371/journal.pcbi.1006692 (PMC6364703; doi:10.1371/journal.pcbi.1006692)

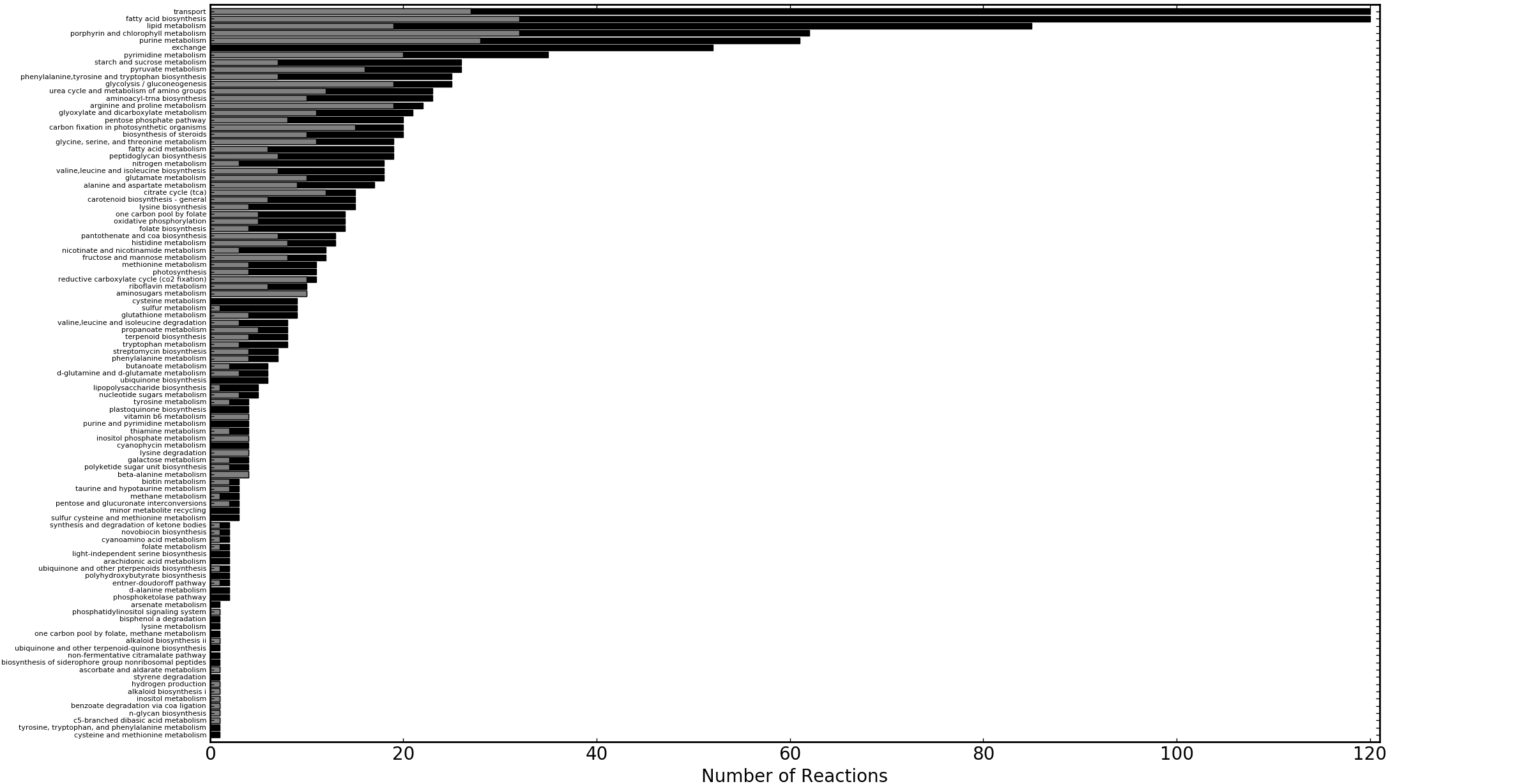

Supplement: S1 Fig — (PNG) [file pcbi.1006692.s008.png]
